# Causes, clinical findings and therapeutic options in chylomicronemia syndrome, a special form of hypertriglyceridemia

**DOI:** 10.1186/s12944-022-01631-z

**Published:** 2022-02-10

**Authors:** György Paragh, Ákos Németh, Mariann Harangi, Maciej Banach, Péter Fülöp

**Affiliations:** 1 Division of Metabolic Diseases, Department of Internal Medicine, University of Debrecen Faculty of Medicine, Nagyerdei krt. 98, Debrecen, H-4032 Hungary; 2 Department of Hypertension, WAM University Hospital in Lodz, Medical University of Lodz, Lodz, Poland; 3 Polish Mother’s Memorial Hospital Research Institute (PMMHRI), Lodz, Poland

**Keywords:** Chylomicronemia syndrome, Lipoprotein lipase, apoC3 inhibitor, MTP inhibitor, Angiopoietin-like protein-3

## Abstract

The prevalence of hypertriglyceridemia has been increasing worldwide. Attention is drawn to the fact that the frequency of a special hypertriglyceridemia entity, named chylomicronemia syndrome, is variable among its different forms. The monogenic form, termed familial chylomicronemia syndrome, is rare, occuring in 1 in every 1 million persons. On the other hand, the prevalence of the polygenic form of chylomicronemia syndrome is around 1:600. On the basis of the genetical alterations, other factors, such as obesity, alcohol consumption, uncontrolled diabetes mellitus and certain drugs may significantly contribute to the development of the multifactorial form. In this review, we aimed to highlight the recent findings about the clinical and laboratory features, differential diagnosis, as well as the epidemiology of the monogenic and polygenic forms of chylomicronemias. Regarding the therapy, differentiation between the two types of the chylomicronemia syndrome is essential, as well. Thus, proper treatment options of chylomicronemia and hypertriglyceridemia will be also summarized, emphasizing the newest therapeutic approaches, as novel agents may offer solution for the effective treatment of these conditions.

## Introduction

Showing high inter-individual variations, hypertriglyceridemia may affect about one third of the population [1]. Ranging from mild and moderate to severe forms, hypertriglyceridemia increases cardiovascular risk and predisposes to acute pancreatitis. Triglycerides are transported in the circulation in chylomicrons that carry dietary (exogenous) fat and very-low density lipoproteins (VLDL) that contain liver-related (endogenous) lipids. Regardless of their origin, triglycerides are hydrolyzed by the endothelium-bound lipoprotein lipase (LPL), which is a key component of the clearance of the triglyceride-rich lipoproteins [2]. A significant portion of the patients experience significantly elevated chylomicron concentrations, thus increased serum triglyceride levels concomitant with elevated serum cholesterol levels.

Chylomicronemia syndrome (CS) may be due to very rare monogenic mutations in the genes encoding the LPL enzyme or its regulators leading to familial chylomicronemia syndrome (FCS) and with the phenotypical appearance of Fredrickson type 1 hyperlipoproteinemia. More commonly, chylomicronemia is a result of clustering multiple genetic variants coexisting with one or more secondary hypertriglyceridemia-aggravating factors leading to multifactorial chylomicronemia syndrome (MFCS, Frecrickson type 5 hyperlipoproteinemia). A third and uncommon form of chylomicronemia syndrome is familial partial lipodystrophy (FPLD) [2]. It is important to mention, that CS may also develop on the basis of autoimmunity (autoimmune hyperlipidemia) and in patients with glucose-6-phosphate deficiency [2].

**Fig. 1:**
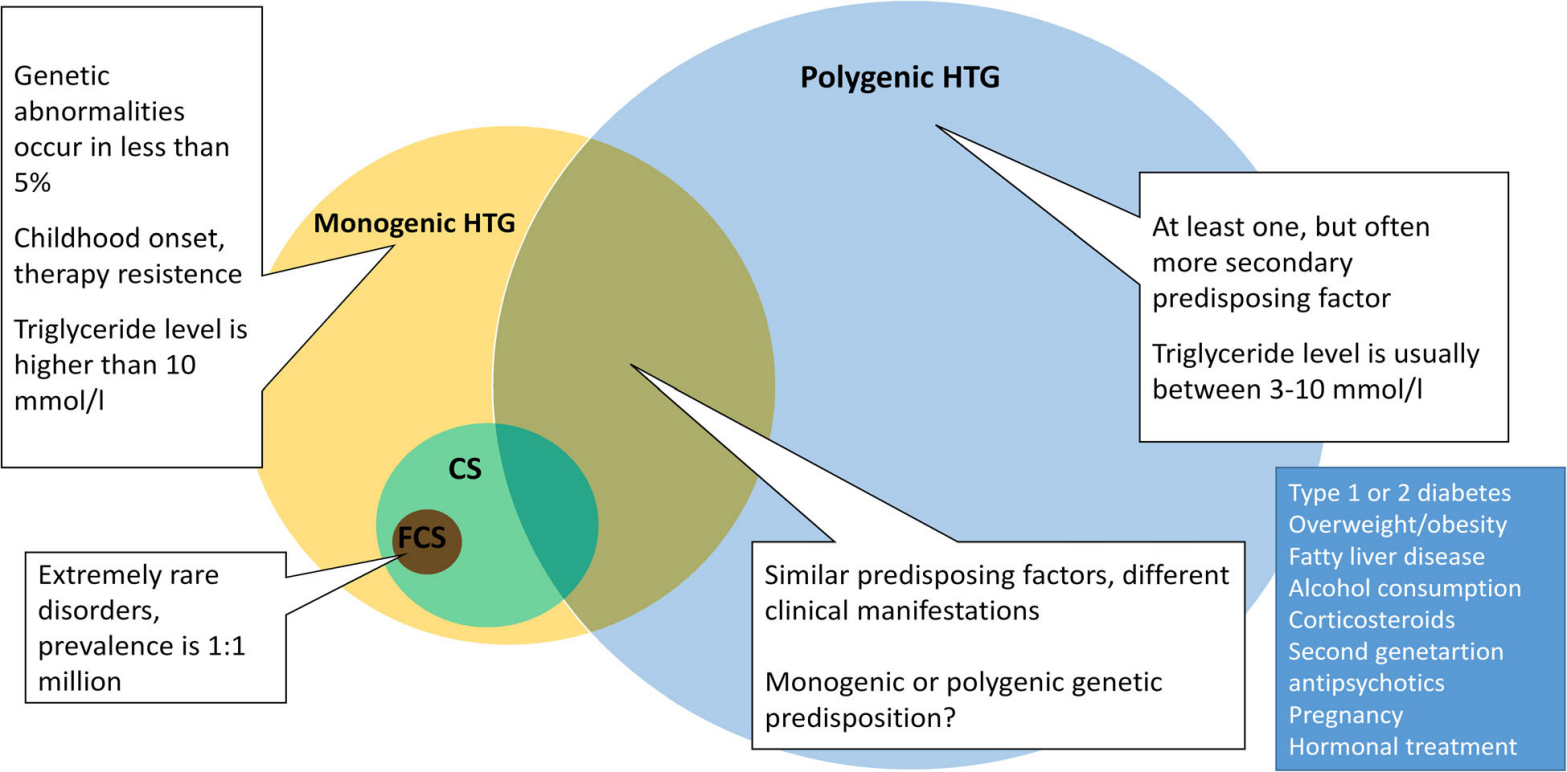
Differential diagnosis of hypertriglyceridemias. Differential diagnosis of elevated triglyceride levels and separation of monogenic and polygenic forms can be challenging in the everyday clinical practice. Secondary forms should be excluded, then characteristics of the monogenic form have to be assessed including childhood onset, resistance to therapy, and severe hypertriglyceridemia exceeding 10 mmol/l. Only a small portion of patients with hypertriglyceridemia present chylomicronemia, and, from these, FCS is a rarity. It should also be noted, that genetic and environmental factors may modify the phenotype and the clinical features of any form

Additionally, hypertriglyceridemia may also be due to another polygenic origin, apperaring as Fredrickson type 4 hyperlipoproteinemia, which is characterized by excess production of VLDL from the liver and may coexist with other co-morbidities. Familial dysbetalipoproteinemia, characterized by homozygous apolipoprotein (apo) E2 mutations, is also associated with significant elevations in triglyceride and cholesterol levels in the serum. In turn, secondary forms of hypertriglyceridaemia are far more common, in which, increased triglyceride concentration is caused by poorly controlled type 2 diabetes mellitus, hypothyroidism, nephrotic syndrome or administration of certain drugs (Fig. 1) [3]. Therefore, finding the exact cause behind hypertriglyceridemia may be a significant challenge to the clinician. In this paper, we focused on chylomicronemia especially from the clinical point of view and discussed the newest etiologic considerations including genetic background. We also detailed the recent therapeutic options, their potential benefits and highlighted the newest developments.

### Lipid metabolism

The amount of circulating fats depends on the balance between the endogenous and exogenous lipid metabolism. Exogenous lipid metabolism refers to the breakdown of dietary lipids, followed by the absorption in the small intestine and conversion to chylomicrons. Entering the endogenous metabolism, chylomicron loses a significant portion of its triglyceride content due to the activity of LPL anchored to the vessel wall, with the triglycerides hydrolyzed into fatty acids and monoglycerides [4]. Chylomicron remnant, which is poorer in triglycerides, is taken up by the remnant receptors of the hepatocytes and may thus enter endogenous lipid metabolism. Triglyceride and other lipid components are attached to the apolipoprotein (apo)B100 by the microsomal triglyceride transfer protein (MTP), forming VLDL. Once in the circulation, VLDL is also degraded by the LPL yielding intermediate density lipoprotein (IDL), that may further be converted to low-density lipoprotein (LDL) by the hepatic lipase [5].

### Factors influencing lipoprotein lipase activity

**Fig. 2:**
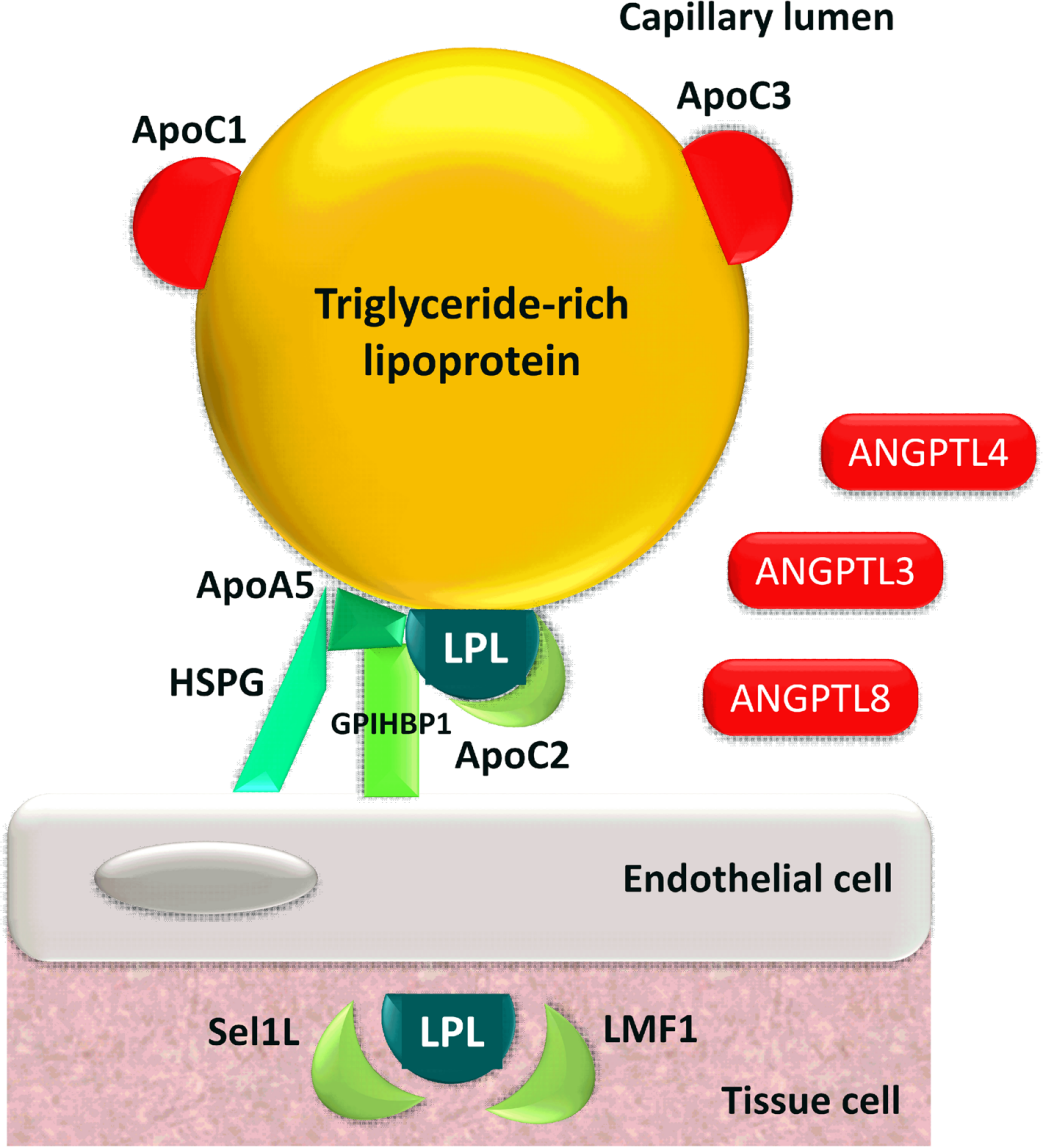
Structure of the lipolytic complex. Lipoprotein lipase (LPL) plays a key role in lipid metabolism. Apolipoprotein (apo) C2 serves as an activator of the enzyme, while lipase maturation factor-1 (LMF1) plays a role in the formation of the proper structure of LPL and regulates its expression. Glycosylphosphatidylinositol-anchored high density lipoprotein-binding protein 1 (GPIHBP1) mediates the transport of LPL into the capillary endothelial cells and anchores it on their surface. ApoA5 stabilizes the LPL-apoC2 complex by promoting endothelial surface binding of triglyceride-rich lipoproteins to heparan sulfate proteoglycan (HSPG). ApoC3 and angiopoietin-like proteins (ANGPTL) 3, 4 and 8 inhibit the activity of LPL

Influenced by a number of cofactors, LPL plays a key role in both the exogenous and the endogenous lipid metabolism. ApoC2 serves as an activator of the enzyme [6–8], hence lack of apoC2 leads to a curbed LPL function. Lipase maturation factor-1 (LMF1) plays a role in the formation of the proper structure of LPL and regulates its expression [9]. As LPL is mainly synthetized in white adipose tissue and skeletal and cardiac muscle, glycosylphosphatidylinositol-anchored high density lipoprotein-binding protein 1 (GPIHBP1) plays a crucial role in its proper function, mediating the transport of LPL into the capillary endothelial cells and anchoring it on their surface [10]. ApoA5 stabilizes the LPL-apoC2 complex by promoting endothelial surface binding of triglyceride-rich lipoproteins to heparan sulfate proteoglycan (HSPG) [11, 12]. ApoE regulates the clearance of the triglyceride-rich lipoproteins, while apoC3 [13] and angiopoietin-like proteins (ANGPTL) 3, 4 and 8 [14–23] inhibit the activity of LPL. The combined effect of these factors sets the actual LPL activity, which determines the extent of triglyceride degradation and thus has a significant impact on the development of hypertriglyceridemia (Fig. 2) [24].

### Clinical features of hypertriglyceridemia

Previous studies have shown that familial chylomicronemia syndrome, in most cases, is a result of a genetic-related alterations in LPL function [25–28]. Loss-of-function mutations lead to a significant reduction in the degradation of triglyceride-rich molecules as chylomicron and VLDL, resulting in severe hypertriglyceridemia. The serum in these patients often appears to be opalescent or even milky, while accumulation of the triglycerides in the skin elicits a local inflammatory response [29, 30]. This presents as a yellowish prominent papule filled with lipid-containing macrophages and surrounded by a red yard, called eruptive xanthoma [31]. The fatty acid pattern of chylomicron (CM) and diabetic eruptive xanthoma triglycerides are similar based on a correlative biochemical, histochemical and electron microscopic study supporting the theory of the plasma lipoprotein origin of xanthoma lipids [32]. Ophthalmological examination may reveal lipemia retinalis, which is the appearance of white, discolored fundus veins and salmon pink areas. This condition, however, is not associated with visual impairment [29, 30]. On the other hand, infiltrating macrophages and Kupffer cells take up large amounts of chylomicrons in the liver, leading to hepatomegaly. A similar phenomenon can also be observed in the spleen, as well. It is important to emphasize, that these lesions are reversible and proper treatment of hypertriglyceridemia leads to their disappearance [29].

The most serious clinical complication in hypertriglyceridemia and FCS is the development of acute pancreatitis with a potential mortality of 5–6% [33–37]. Previous studies indicated that 15% of the patients with severe hypertriglyceridemia had a history of acute pancreatitis [38] with a 5-year pancreatitis rate being greater than 3.5% [39]. The absolute and relative risks of developing acute pancreatitis increase slightly above triglyceride levels of 10 mmol/l [3, 40, 41], and then even more when serum triglycerides exceed 20 mmol/l [38]. This is due to the fact that pancreatic lipase enters the capillaries of the pancreas during the state of hypertriglyceridemia. As a result, partial lipoprotein lipolysis is initiated and the release of free fatty acids damages directly the cells and activates trypsinogen leading to local inflammation and pancreatic cell necrosis [3, 34, 41]. The risk of pancreatitis can significantly be reduced by lowering triglyceride levels, suggesting that significant triglyceride accumulation is a potential life-threatening condition impairing quality of life. Mutations in the genes regulating LPL activity may modify the hydrolysis of the chylomicron particles and usually result in less severe phenotypes than that of the mutations in the *LPL* and *apoC2* genes [27, 42].

### Epidemiology of chylomicronemia syndrome

**Fig. 3:**
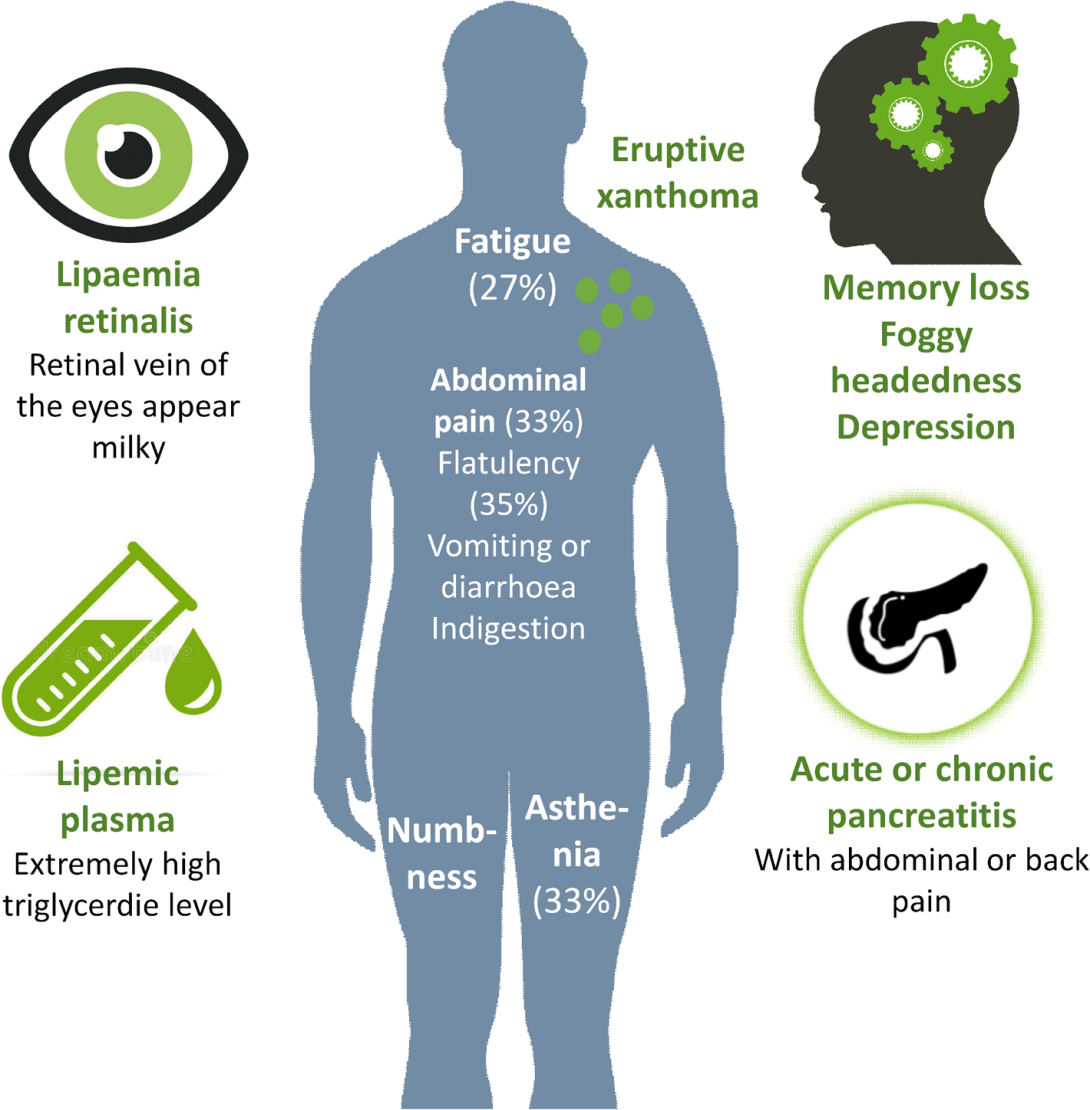
Common symptoms of familial chylomicronemia. Presence of eruptive xantomata and lipemic plasma with milky appearance are characteristic, although not specific features of familial chylomicronemia. Ophthalmological examination may reveal lipemia retinalis. Other symptoms may also be present including abdominal pain, flatulency indigestion and foggy headedness, with acute pancreatitis being the most severe complication

Based on the circulating triglyceride levels, hypertriglyceridemia can generally be divided into two categories: (i) a mild to moderate form in which fasting triglyceride levels are between 2 and 10 mmol/l and (ii) a severe form in which triglyceride levels are above 10 mmol/l [43]. Regardless of the clinical consequences, chylomicronemia is described as an abnormal accumulation of chylomicrons in the plasma for 12–14 h after a meal. Of note, in individuals with undisturbed lipid metabolism, chylomicrons disappear from the serum 2–3 h after a meal [3, 26–28, 44]. If chylomicrons are present in the serum in the fasting state, triglyceride levels exceeding 10 mmol/l may be detected. A typical finding in CS is highly lipemic fasting blood showing a creamy white layer on its surface. After an overnight storage of the blood sample or after centrifugation, this white chylomicron layer can be detected above the other components of the plasma. In CS, at least one of the following clinical findings or symptoms is present: eruptive xanthoma of the trunk and limbs, lipemia retinalis, recurrent abdominal pain, acute or recurrent pancreatitis, and hepatosplenomegaly ***(***Fig. 3***)*** [26, 27, 45, 46].

About 95% of chylomicronemia is of multifactorial origin (MFCS), while 5% has monogenic background with autosomal recessive inheritance (FCS) with rare cases of FPLD. The polygenic/multifactorial form is estimated to occur in 1 in every 600 individuals, while the prevalence of monogenic chylomicronemia is 1:1,000,000 [47]. The latter prevalence is similar to that of the homozygous form of familial hypercholesterolemia. More than 90% of monogenic chylomicronemia is due to mutations in the *LPL* gene [25]. Indeed, more than 114 *LPL* gene mutations have been described so far [25, 26, 48–55]. Other mutations influencing LPL activity are much less common, such as mutations in the genes of the regulators of LPL including *apoC2, apoA5, LMF-1,* and *GPIHBP1* [28, 42].

Assessment of the genetic background of MFCS has been slightly altered in the last few years [56, 57]. According to the recent data, FCS is bi-allelic, due to homozygous or compound heterozygous mutations in the genes of LPL, ApoC2, GPIHBP1, LMF1 that cardinally affect the catabolic efficacy of LPL complex. However, causal involvement of mutations of ApoA5 in FCS is ambiguous, since ApoA5 may not be an essential cofactor for LDL activity. Therefore, its deficiency may not be sufficient to abolish LPL activity [58]. Furthermore, in patients diagnosed with MFCS, causes include mono-allelic pathogenic variants in one of the above 5 genes associated with high polygenic risk [48]. Indeed, a recent study found that some patients diagnosed with FCS exhibiting LPL activity deficiency harbored mutations only in heterozygosity, which raises the question of the involvement of concomitant SNPs, new genes or environmental factors in the manifestation of FCS [59]. It must be underlined that CS cases with monogenic origin are rare, and polygenic risk is the most common and important susceptibility factor for CS [56].

On the basis of complex genetic susceptibility affecting heterozygous variants of the above mentioned genes and/or common polymorphisms of other genes with smaller impact on LPL function, multifactorial chylomicronemia syndrome is usually provoked by secondary factors including obesity, alcohol consumption, poorly controlled diabetes mellitus, hypothyroidism, nephrotic syndrome and many commonly used medications as estrogen, estrogen receptor antagonists, corticosteroids, thiazide diuretics, beta-blockers, retinoids, resins, antipsychotics, antidepressants and antiretroviral agents [3]. In this form, concentrations of a wide range of lipoprotein fractions are elevated, such as apoB48-containing chylomicron remnants, apoB100-containing VLDL, VLDL remnants and IDL, together with decreased high-density lipoprotein (HDL) levels [60]. Postprandial accumulation of both apoB48-containing and hepatic apoB100-containing triglyceride-rich lipoprotein remnants is considered to be pro-atherogenic. It is important to emphasize that multifactorial chylomicronemia, as a marker of postprandial lipemia, is also considered to be a pro-atherogenic metabolic condition [60].

In about 30% of patients with CS, neither recessive nor heterozygous variants, or frequent single nucleotide polymorphisms (SNP) can be detected. These are called unidentified chylomicronaemias. Regardless of the origin, the risk of life-threatening pancreatitis is increased. Cardiovascular risk may not be significantly affected in the monogenic form, while it is increased in patients carrying multifactorial CS [25]. A recent multicenter study evaluated the risk of cardiovascular complications retrospectively, based on a 10-year comparative follow-up of FCS versus MFCS patients. Twenty-nine FCS and 124 MFCS patients, with genetic diagnosis, in 4 lipid clinics were matched with 413 controls were enrolled. The ischemic risk (risk of hospitalization for ischemic cardiovascular disease) was lower in FCS than in MFCS (HR = 3 *p* < 0.05). It must be noted that they could not detect any major adverse cardiovascular events (acute myocardial infarction, cerebral ischemia, stroke, not specified as hemorrhage or infarction, transient cerebral ischemic attacks, and related syndromes) during the 10-year follow-up, underlining the minor risk of cardiovascular complications in FCS [61]. Monogenic chylomicronemia typically occurs in neonates and young children or in the adolescence, at the latest [26, 44]. Besides the above mentioned, hypertriglyceridemia with genetic origin may either have autosomal dominant or polygenic inheritance involving inactivating *LPL* gene mutations or variants modifying apoB metabolism and the diseases may also be associated with chylomicronemia syndrome [62].

The third, uncommon form of CS is FPLD, which is a heterogenous group of rare conditions characterized by adipose tissue maldevelopment and lipoatrophy. The condition is characterized by limited capacity of adipose tissue to store fat leading to subsequent metabolic abnormalities, such as insulin resistance, liver steatosis and severe hypertriglyceridemia [2, 63, 64]. Of the several forms of FPLD, the two most common are the Dunnigan (type 2) and Köbberling (type 1) varieties. Mutations of the LMNA and the PPARG genes are usually found in patients with the Dunnigan form, while no mutations in single genes have been identified in the Köbberling phenotype, although polygenic background has been confirmed [65]. It is important to emphasize that the latter form shares many features with metabolic syndrome; however, careful physical examination of the fat distribution may help a lot. Familial dysbetalipoproteinemia (Fredrickson type 3 hyperlipoproteinaemia) can be another possible cause of chylomicronemia if it co-exists with a secondary form of hypertriglyceridemia such as pregnancy or administration of oral contraceptives [66].

Other conditions, such as lupus erythematosus, Sjögren’s syndrome or multiple myeloma may also lead to chylomicronemia and hypertriglyceridemia [2]. Antibodies to LPL, VLDL or GPIHBP1 are reported to develop in these patients, resulting in autoimmune hyperlipidemia [67].

Identification and proper control of conditions provoking or unmasking multifactorial chylomicronemia are of major importance, as these factors are the most common causes of hypertriglyceridemia in the everyday practice [46].

### Diagnosis of chylomicronemia syndrome

In general, distinction between the two forms may be made on the basis of clinical symptoms, laboratory results, and genetic tests; however, the proper diagnosis may be hard to establish due to the various factors that influence circulating triglyceride concentration.

### Monogenic form (FCS)

Symptoms begin in early childhood and the clinical abnormalities mentioned earlier as abdominal pain of unknown origin, eruptive xanthomas, hepatosplenomegaly, and recurrent pancreatitis are common [26, 27, 45, 46]. Previous studies have shown that a 1.1 mmol/l elevation in the serum triglyceride level increases the risk of acute pancreatitis by 4% [3, 68]. This association has also been observed in prospective studies in moderate hypertriglyceridemia [69]. In this form, neurological symptoms as irritability, memory impairment, dementia, and depression are often observed [70]. Abdominal complaints result in decreased food intake, deteriorating quality of life, and decreased daily activity [71, 72]. Plasma triglyceride is usually higher than 10 mmol/l and the triglyceride:cholesterol ratio is above 2.2, with apoB100 levels exceeding 100 mg/dl. Lipoprotein lipase activity decreases drastically 10 min after intravenous administration of heparin.

### Polygenic chylomicronemia (MFCS)

In addition to the high triglyceride levels, presence of other triggering factors may be detected in this form, as detailed above. Moderate hypertriglyceridemia and a loss-of-function mutation in apoA5 are commonly observed [73]. A previous genome wide association study (GWAS) proved that several single nucleotide polymorphisms (SNPs) associated with hypertriglyceridemia in normolipidemic samples, including SNPs in the genes of APOA5, TRIB1, TBL2, GCKR, GALNT2 and ANGPTL3, were significantly associated with hyperlipoproteinemia Fredrickson types 2B, 3, 4 and 5 [56]. The findings of the GWAS studies reinforce that the genetic contribution to most of the hyperlipoproteinemia phenotypes is complex, but also suggest that additional genes or non-genetic factors may still play an important role. It is not surprising that Pplasma triglyceride levels are much more variable and more sensitive to diet and fibrate therapy compared to monogenic forms.

### FCS screening

The score system recommended by Moulin et al. seems to be promising in screening for FCS [74] (Table 1). Based on the information to date, recent knowledge regarding the separation of monogenic and polygenic chylomicronemias is summarized in Table 2.

**Table 1:** Scoring of familial chylomicronemia syndrome, according to Moulin et al.

	Score
1. Fasting TGs > 10 mmol/L for 3 consecutive blood analysis	+ 5
Fasting TGs > 20 mmol/L at least once	+ 1
2. Previous TGs < 2 mmol/L	-5
3. No secondary factor (except pregnancy and ethinylestradiol)	+ 2
4. History of pancreatitis	+ 1
5. Unexplanied recurrent abdominal pain	+ 1
6. No history of familial combined hyperlipidaemia	+ 1
7. No response (TG decrease < 20%) to hypolipidaemic treatment	+ 1
8. Onset of symtoms at age:	
- < 40 years	+ 1
- < 20 years	+ 2
- < 10 years	+ 3

> 10: FCS very likely

< 9 FCS unlikely

< 8 FCS very unlikely

**Table 2:** Characteristics of different types of primary chylomicronemia syndrome

	Polygenic (multifactorial chylomicronemia - MCS)	Monogenic (familial chylomicronemia – FCS)
Frequency	95%	5%
Clinical findings	Chylomicronemia, eruptive xanthoma, lipemia retinalis, abdominal pain, recidiv pancreatitis, hepatosplenomegaly	Chylomicronemia, eruptive xanthoma, lipemia retinalis, abdominal pain, recidiv pancreatitis, hepatosplenomegaly
Onset of the symtoms	In adult	Early age, childhood and adolescent
Secondary factors which initiate the symptoms	High amount fatty food intake, alcohol, diabetes mellitus, obesity, hypothyreosis, metabolic syndrome, nephrotic syndrome, drugs (oestrogen, corticosteroid, retinoid, beta-blockers, thiazide, resin, second generation antipsychotic and antiretroviral agents)	–
Plasma left overnight is milky with a creamy layer on top	+	+++
Triglyceride level	< 10 mmol/l, or less	> 10 mmol/l
Plasma apoB< 100 mg/dl		+
Triglyceride/Cholesterol ratio > 2.2 mmol/l (5 mg/mg)	+	+++
I.v. 50 U/kg heparin induced lipoprotein lipase activity	After 10 min normal, After 60 min reduced	very low
Effect of tradicional lipid lowering therapy (fibrate, acidum nicotinicum, omega-3 fatty acid)	Moderate effect	no effect
Secondary risk factor treatment improve the symptoms	++++	
Genetic background	Single copy or monoallelic (i.e. heterozygous) rare variants in LPL, APOC2, APOA5, LMF1 and GPIHBP1 gene and polygenic risk from common variants associated with TG levels	Frequently: LPL, ApoC2 mutations Rare cases: ApoA5, GPIHBP1, LMF1

### Treatment of chylomicronemia

The key components of CS management are focusing primarily on pancreatitis prevention and secondarily on cardiovascular risk lowering including prevention of nonalcoholic steatohepatitis (NASH) and type 2 diabetes mellitus.

### Lifestyle and diet

Non-pharmacologic interventions including cessation of aggravating factors and introducing very-low fat diet that contain less fat than 15% of the total calories is recommended, which may significantly reduce clinical signs and symptoms. In FCS patients dietary fat restriction to less than 5 to 10% of calories consumed and medium chain triglyceride may help to decrease chylomicron formation [2]. At the same time, diet helps to normalize body weight, however, sustaining such diet on a long-term basis is a huge challenge, especially in young individuals. Overweight and diabetic patients benefit from weight reduction together with high-fiber and low-carbohydrate diet. Discontinuation of alcohol helps to lower VLDL production and reduces pancreatitis incidence, while removal of potential triggering drugs and treatment of those conditions that are associated with hypertriglyceridemia are of major importance, as well [26, 44].

### Drugs

Lifestyle treatment alone is usually not enough, thus it should be supplemented with medication. Of the currently available drug treatments, the most commonly used are fibrates, nicotinic acid and its derivatives, and statins. Their lipid lowering efficacy in FCS, however, unfortunately lags behind that desired [75, 76].

### Fibrates

Via the peroxisome proliferator activating receptor (PPAR)-α, fibrates inhibit apoC3 expression and hepatic synthesis of the triglyceride-rich VLDL particles; however, they do not have an impact on the level of the intestinum-derived chylomicrons. Fibrates also enhance LPL-mediated lipolysis by reducing apoC3 levels, which is an inhibitor of LPL activity [3]. Fibrates possess a triglyceride lowering effect of up to 50% [3, 77–79]. In individuals with mixed dyslipidemia that is associated with the release of chylomicrons and VLDL particles, fibrates reduce triglyceride levels by decreasing the number of VLDL particles. In FCS, characterized by loss-of-function LPL gene mutation, the response to treatment is very modest due to the lack of the enzyme activity.

A previous study demonstrated the efficacy and safety of pemafibrate, a novel selective peroxisome proliferator-activated receptor α modulator (SPPARMα) in three patients with severe hypertriglyceridemia [80]. However, a recent case report verified a modest efficacy of pemafibrate in a woman with LPL deficiency [81]. Further studies are needed to clarify the potential therapeutic role of pemafibrate in FCS.

### Niacin

Niacin decreases lipolysis and free fatty acid release from adipose tissue by inhibiting hormone-sensitive triglyceride lipase [82]. It also inhibits diacylglycerol O-acyltransferase 2 [3, 77, 79, 83], which also contributes to curbed VLDL production. Indeed, the triglyceride lowering effect of niacin is between 5 and 35% [84]. Additionally, niacin reduces the degradation of apoA1-containing proteins and increases the expression of PPAR- gamma [85]; however, the common side effects seriously limit its use.

### ω-3 fatty acids

Intake of high doses of *ω*-3 fatty acids (4–6 g eicosapentaenoic acid (EPA) or doxosahexaenoic acid (DHA) daily) inhibits VLDL production, reduces the chylomicron size [86], enhances systemic lipolysis and promotes chylomicron plasma clearance by inhibiting apoC3 [87–90]. It also reduces apoC3 production [91] and increases chylomicron remnant clearance [86]. It can exert a triglyceride-lowering effect of up to 21% [92]. Recent data also indicate that icosapent ethyl (IPA), a purified derivative of EPA, is also effective in lowering triglyceride levels, and, even more importantly, reduces cardiovascular risk [93].

### Diacylglycerol O-acyltransferase 1 (DGAT1) inhibitors

Localized in the endoplasmic reticulum, DGAT1 is a key enzyme in triglyceride synthesis showing high expression in the intestine, liver and white adipose tissue [94, 95]. Unfortunately, previous studies indicated that, the use of the DGAT1 inhibitor AZD7687 had provoked severe nausea, vomiting, and intolerable diarrhea [96–98], therefore DGAT1 inhibitor development has been halted.

### ApoC3 inhibition

ApoC3 is produced by the liver and small intestine and is found mainly in triglyceride-rich lipoproteins, such as chylomicrons and VLDL. Physiologically, apoC3 inhibits LPL and hepatic lipase activities as well as the hepatic uptake of triglyceride-rich lipoproteins [99]. Inhibition of apoC3 expression significantly increases LPL activity and decreases triglyceride production [100]. Mutations associated with decreased apoC3 have been found to reduce the risk of major cardiovascular events in addition to decreasing plasma triglyceride levels [101]. The apoC3 antisense oligonucleotide inhibits its protein production and was found to lower apoC3 levels by 77% and triglyceride levels by 43% in healthy subjects. This effect persisted for 4 weeks after discontinuation of the drug and no serious side effects were observed [102]. Another study showed an 80% apoC3 and 70% triglyceride-lowering effect in monogenic chylomicronemia with LPL deficiency [103]. In phase 2 studies, the second-generation apoC3 inhibitor volanesorsen had a 31–71% triglyceride-lowering effect [104].

In the phase 3, randomized, double-blind, placebo-controlled APPROACH study, patients with familial chylomicronaemia syndrome received 300 mg volanesorsen administered subcutaneously once a week. The apoC3 inhibitor showed a very potent and sustained effect, decreasing serum triglyceride levels by 77%. No recurrent pancreatitis occurred, abdominal pain was reduced, and no side effects related to liver or kidney function were observed. The most common side effect was a local reaction at the injection site and, in 5 cases, a decrease in platelet count, which necessitated discontinuation of treatment [105]. The beneficial effects of volanesorsen were confirmed in MFCS patients by the COMPASS study as well, in which a 72% reduction in the triglyceride levels was observed in the patients treated with the apoC3 inhibitor. There was a significant reduction in the incidence of pancreatitis and the most common side effect was a local skin reaction. However, no significant difference in platelet count was observed. The study demonstrated that the apoC3 inhibitor treatment may have a significant triglyceride-lowering effect in hypertriglyceridaemia with or without familial chylomicronemia syndrome [106]. Based upon these results, the use of volanesorsen was approved in the European Union for the treatment of FCS in adults [107].

### LPL gene replacement therapy

Alipogene tiparvovec is a kind of adenovirus-associated virus that encodes an increased activity variant of the human *LPL* gene [108–111] and can lower triglyceride levels by 40%. However, due to its transient effect, the drug development was discontinued in 2017.

### Angiopoietin-like protein 3 (ANGPTL3) inhibition

ANGPTL3 is mainly produced by the liver after meals reversibly inhibiting LPL activity [14]. Several studies have demonstrated that recombinant ANGPTL3 inhibits LPL activity in vitro [15–19]. ANGPTL3 is much more effective in the presence of ANGPTL8 and previous studies have shown that the effect of increased ANGPTL3 production on plasma triglyceride levels was significantly reduced in the absence of ANGPTL8 [21, 22, 112]. ANGPTL3 production in the liver is mediated by the oxidized sterol-activated liver X receptor (LXR) [113, 114] and LXR agonists increase circulating triglyceride levels [115]. Expression of ANGPTL3 is inhibited by PPAR-γ agonists, statins, insulin, leptin, thyroid hormone, and lipopolysaccharides [116–121]. Patients with mutations in the *ANGPTL3* gene had decreased total cholesterol, LDL, triglyceride levels, and HDL levels [23]. *ANGPTL3* gene mutated mice express decreased triglyceride, total cholesterol and unesterified fatty acid concentrations in plasma, which can be reversed following administration of ANGPTL3-expressing adenovirus [122]. Also, ANGTPL3 antisense oligonucleotide reduces serum cholesterol and triglyceride levels [123] and a monoclonal antibody to ANGPTL3 named evinacumab was also effective in reducing triglyceride and cholesterol levels, as well as HDL and LDL-C and VLDL-C levels in mice [124, 125]. The decrease might be due to increased degradation of LPL-mediated triglyceride-rich lipoproteins [16, 100, 126]. Additionally, vupanorsen, a second generation ANGTPL3 antisense oligonucleotide showed similar effects on apoC3 and triglyceride levels to that of volanesorsen, but was found to provoke less number of adverse effects [127].

### Statins

Inhibiting the hepatic hydroxymethylglutaryl-coenzyme A (HMGCoA) reductase, statins promote the uptake of LDL particles from the circulation. However, no significant impact on LDL concentration was observed in primary CS [101]. Statins also increase the catabolism of the remnants of LDL and chylomicrons, contributing to the reduction of triglyceride levels [128]. Although these drugs cannot reduce chylomicrons or improve severely increased triglyceride levels, based upon the etiology of MFCS, statin therapy is a key player in cardiovascular disease risk reduction.

### MTP inhibitor

MTP binds triglyceride and other lipid components to apoB100 and apoB48 [94, 129–132], therefore inhibiting this process may lead to reduced VLDL and chylomicron formation. The MTP inhibitor lomitapide is currently not approved in FCS, although provides a 30–40% triglyceride lowering effect in homozygous familial hypercholesterolemia [94, 129–131]. Gastrointestinal side effects, however, are common, including nausea, vomiting and diarrhea. The incidence of these side effects may significantly be reduced with increasing the dose gradually [129, 133, 134]. It also has to be mentioned that the use of MTP inhibitors increases liver fat content and the risk of developing steatohepatitis and fibrosis. Recurrence of pancreatitis could also be mitigated, due to the 60–70% reduction in triglyceride concentration with lomitapide therapy in monogenic chylomicronemia [130].

### Other preparations

CAT-2003 is a novel conjugate of EPA and niacin that is hydrolyzed by fatty acid amine hydrolase to release EPA in cells. CAT-2003 blocks the maturation of sterol regulatory element-binding proteins (SREBP)-1 and − 2, inhibiting the production of the proprotein convertase subtilisin/kexin type 9 (PCSK9), which leads to an increased number of LDL receptors on the cellular surface and decreased serum cholesterol and triglyceride levels [135]. Zimmer et al. have found that CAT-2003 reduced prandial triglyceride levels by 30% and postprandial concentrations by 90% [136]. In patients with familial hyperlipoproteinemia type 1, the efficacy and safety of CAT-2003 are recently studied including its effects on fasting levels of total and chylomicron triglycerides and postprandial total and chylomicron triglyceride clearance, as well as plasma non-HDL lipoprotein levels, and the frequency of adverse events. This study may also confirm the role of CAT-2003 in the treatment of hypertriglyceridemia [135, 137].

### Plasmapheresis

In severe chylomicronemia and acute pancreatitis, plasmapheresis may be used to rapidly reduce the serum concentrations of triglyceride-rich lipoproteins and manage hypertriglyceridemia-associated pancreatitis [138–146]. Acutely, when oral therapy cannot be used, plasmapheresis is one way to reduce triglyceride level [147]. Comparing plasmapheresis with conservative therapy, it was found that plasmapheresis was not more beneficial than conservative therapy [34, 148, 149]. In uncontrolled chylomicronemia, insulin infusion elicited a greater degree of triglyceride reduction than plasmapheresis [150]. Of note, the risk of hypoglycemia should be addressed during insulin administration. To date, there are no controlled clinical studies that demonstrate the superiority of plasmapheresis on conservative therapy, including dietary restriction, fluid replacement, analgesia, and control of secondary factors. Therefore, at present, some centers only recommend plasmapheresis in severe hypertriglyceridemia associated with pregnancy. It is common practice to perform plasmapheresis when triglyceride level is above 60 mmol/l to prevent the development of acute pancreatitis and hyperviscosity syndrome. Plasmapheresis is also used at a triglyceride value of 40 mmol/l, in case of abdominal complaints and symptoms of hyperviscosity syndrome. Plasmapheresis reduces triglyceride levels without affecting the underlying cause, therefore treatment for the causes of hypertriglyceridemia should also be initiated at the same time.

### Novel aspects of this review

Fredrickson’s classification of hyperlipoproteinemias are still taught and used in several regions of Europe; however, based upon the most recent data, such classification seems to be outdated. Therefore, we aimed to harmonize previous nomenclature with recent knowledge in our review in order to mitigate potential misunderstandings and help daily bedside routine.

Despite some other valuable reviews, we tried to reveal the complexity and the difficulties of the diagnosis of hypertriglyceridemia and chylomicronemia from the clinicians’ point of view. In the light of these challenges, we also aimed to detail the most up-to-date therapeutic options highlighting their practical significance and usability. Additionally, we tried to draw the attention to the fact that some, experimentally fascinating, treatment approaches failed to live up to the initial expectations and necessitated discontinuation of such drug development.

## Conclusions

Chylomicronemias leading to hypertriglyceridemia represent a major clinical burden with the potential development of life-threatening pancreatitis and cardiovascular diseases. Although a very large proportion of these individuals have a multifactorial form of chylomicronemia and hypertriglyceridemia, that may be exacerbated by common conditions such as obesity, diabetes mellitus, alcohol intake and administration of certain drugs, a minor proportion of CS subjects have a monogenic genetic background with severe and persistent chylomicronemia leading to recurrent pancreatitis, cardiovascular complications and fatty liver disease. These underline the importance of the early diagnosis and proper treatment of this patient population. Novel therapeutic agents may offer solution for the effective treatment of severe hypertriglyceridemias including FCS.

## Data Availability

Not applicable.

## Abbreviations

ANGPTL: Angiopoietin-like proteins
apo: Apolipoprotein
CS: Chylomicronemia syndrome
DHA: Doxosahexaenoic acid
DGAT1: Diacylglycerol O-acyltransferase 1
DNA: Deoxyribonucleic acid
EPA: Eicosapentaenoic acid
FCS: Familial chylomicronemia syndrome
FH: Familiar hypercholesterolemia
FPLD: Familial partial lipodystrophy
GPIHBP1: Glycosylphosphatidylinositol-anchored high density lipoprotein-binding protein 1
HDL: High-density lipoprotein
HMGCoA: Hydroxymethylglutaryl-coenzyme A
HSPG: Heparan sulfate proteoglycan
IDL: Intermediate-density lipoprotein
LDL: Low-density lipoprotein
LPL: Lipoprotein lipase
LMF1: Lipase maturation factor-1
MFCS: Multifactorial chylomicronemia syndrome
MTP: Microsomal triglyceride transfer protein
NASH: Nonalcoholic steatohepatitis
PCSK9: Proprotein convertase subtilisin/kexin type 9
PPAR: Peroxisome proliferator activating receptor
SNP: Single nucleotide polymorphisms
SREBP: Sterol regulatory element-binding proteins
VLDL: Very-low density lipoproteins
